# Case Report: Oral and topical chronic administration of THC-rich and CBD-rich cannabis oil as palliative care in a rescued horse with open wound, sarcoid and chronic pain

**DOI:** 10.3389/fvets.2026.1794084

**Published:** 2026-06-02

**Authors:** Rodrigo Zamith Cunha, Eduarda Hausen Kuhn, João Lucas Lopes Medeiros, Giulia Canale Medeiros, Erik Amazonas

**Affiliations:** 1 Department of Veterinary Medical Sciences, Alma Matter Studiorum - University of Bologna, Bologna, Italy; 2 Department of Veterinary Medical Sciences, University of Teramo, Teramo, Italy; 3 Independent Veterinary Practitioner, Santa Catarina, Florianopolis, Brazil; 4 Postgraduate Program in Conventional and Integrative Veterinary Medicine (PPGMVCI), Cannabis Development and Innovation Hub (PODICAN), Federal University of Santa Catarina (UFSC), Curitibanos, Brazil

**Keywords:** appetite and food intake, Cannabidiol (CBD), delta 9 tetrahydrocannabinol, equine medicine, fibroblastic, pain management

## Abstract

Cannabinoid-based therapies have shown analgesic, anti-inflammatory, and wound-healing potential across veterinary species; however, clinical data on long-term use of THC-rich formulations in horses remain scarce. This case report describes the use of combined oral and topical THC-rich and CBD-rich full-spectrum cannabis oils as part of a palliative care strategy in a rescued horse with severe chronic disease. A senior mixed-breed gelding was rescued with a large, chronic ulcerative lesion of the left hind limb, severe malnutrition, non-weight-bearing lameness (AAEP grade 5/5), and refractory pain. Diagnostic workup identified a fibroblastic equine sarcoid complicated by complete suspensory tendon rupture, early osteomyelitis, and chronic joint disease. Conventional medical and surgical options were limited due to poor response, disease severity, and resource constraints. A long-term palliative protocol was initiated using oral THC-rich and CBD-rich full-spectrum cannabis oils (1:1 ratio; 100 mg/mL each) with gradual dose escalation to a target of 0.5 mg/kg of each compound every 12 hours. The same formulation was applied topically to the wound once to twice daily. Treatment duration was 10 months, with concurrent multimodal analgesia as needed. Cannabinoid therapy was associated with sustained improvements in appetite, body condition, pain, and mobility. Marked wound improvement was observed, including reduced granulation tissue, improved epithelialization, and resolution of self-mutilation. No clinically relevant adverse effects or laboratory abnormalities occurred during routine dosing. Transient ataxia and sedation were noted only at high rescue doses near end of life. Despite eventual disease progression and euthanasia due to refractory pain, quality of life was substantially improved for most of the treatment period. This case supports the potential role of combined THC-rich and CBD-rich cannabis oils as a safe and effective adjunct in multimodal palliative care for horses with chronic, refractory conditions. Controlled studies are warranted to define optimal dosing and indications.

## 1 Introduction

The EndoCannabinoid System (ECS) is a cellular signaling network composed of endocannabinoids (such as anandamide, AEA, and 2-arachidonoylglycerol, 2-AG), receptors (primarily Cannabinoid receptor 1 - CB1R - and type 2 - CB2R), and enzymes responsible for synthesis and degradation (including Fatty Acid Amide Hydrolase – FAAH - and Monoacylglycerol Lipase - MAGL) (1). It plays essential roles in regulating mood, sleep, reproduction, pain perception, metabolism, and immune responses (2–6). In veterinary medicine, cannabinoid receptors have been identified in dogs (7), cats (8), and horses (9). Their expression has also been studied in healthy versus diseased tissues in conditions such as feline chronic gingivostomatitis, equine laminitis, and canine atopic dermatitis (8, 10, 11).

The most studied *Cannabis*-derived compounds are cannabidiol (CBD) and delta-9-tetrahydrocannabinol (THC). These molecules are classified as phytocannabinoids and exert distinct biological effects depending on factors such as receptor affinity, tissue distribute on, species, and their relative concentrations within a formulation. CBD demonstrates anxiolytic effects in humans (12) and dogs (13, 14), anti-tumoral properties in dogs (15) and humans (16), anti-inflammatory actions in humans (17) and horses (18, 19), and promotes analgesia in horses (20–22). The benefits of cannabinoids as palliative support in human oncology are well documented (23–28).

Across veterinary species, cannabinoids have shown therapeutic potential with favorable safety and tolerability profiles. In horses, particularly, CBD have demonstrated efficacy and safety across a broad dosing range (18, 20, 22, 29, 30). Despite THC’s therapeutic benefits, published data on its veterinary use remain scarce, in equids there is only a single report of toxicosis in donkeys (31) that consumed cannabis plants ad libitum. To the authors’ knowledge, there are no published clinical reports describing THC or THC-rich formulations in horses.

Equine sarcoids are common cutaneous tumors characterized by local invasiveness, high recurrence rates, and challenging clinical management (32). Chronic pain control in horses also remains difficult, as many conventional analgesics have limited long-term efficacy, narrow safety margins, and significant adverse effects (33). In severe cases, pain and inflammation may become refractory to standard therapeutic approaches. The therapeutic effects of cannabinoids on skin inflammation (34, 35), wound healing (36, 37), and pain modulation (38) are well described in the literature, making them a promising adjunctive option in complex or treatment-resistant cases.

The aim of this case report is to describe a long-term multimodal clinical approach using cannabinoids (THC and CBD) in a rescued horse affected by a wound/sarcoid and joint disease, as part of a palliative care strategy for pain management, inflammation control, and wound-healing enhancement. The case referred to specialized care after poor response to conventional treatments, progressive weight loss, and increasing difficulty in managing the sarcoid, for which surgery and electrochemotherapy were not viable due to geographic location and financial constraints.

## 2 Case description

**Figure 1:**
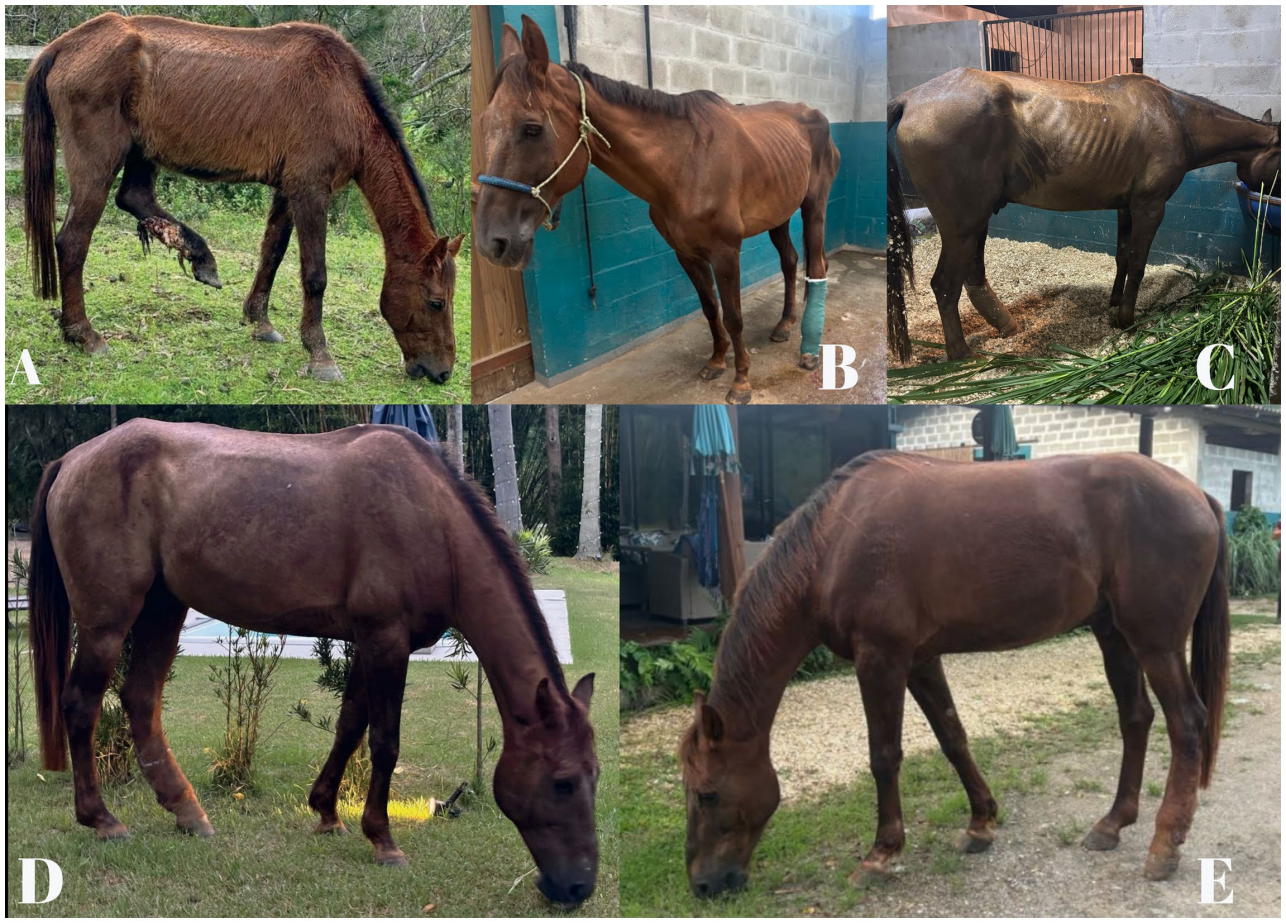
Sequential photographic documentation of body condition score (BCS) evolution in a rescued horse from October 2024 to July 2025, illustrating progressive recovery from severe under conditioning to an improved and stabilized body condition. **(A)** Day of rescue (October 2024), showing severe emaciation and markedly reduced BCS. **(B)** November 2024, prior to initiation of cannabinoid therapy, with persistent poor body condition. **(C)** January 2025, early improvement in BCS following clinical stabilization. **(D)** April 2025, continued and substantial recovery of body condition. **(E)** July 2025, near-normalized body condition with restored muscle mass and overall improved BCS.

A senior mixed-breed gelding, weighing approximately 390 kg and with a body condition score (BCS) of 1/5, was rescued after being found abandoned in a vacant lot. The horse showed clear signs of neglect, including severe malnutrition, generalized muscle atrophy, poor coat quality, and reduced appetite, although he was still observed grazing intermittently (Figure 1A).

### 2.1 Initial clinical examination

**Figure 2:**
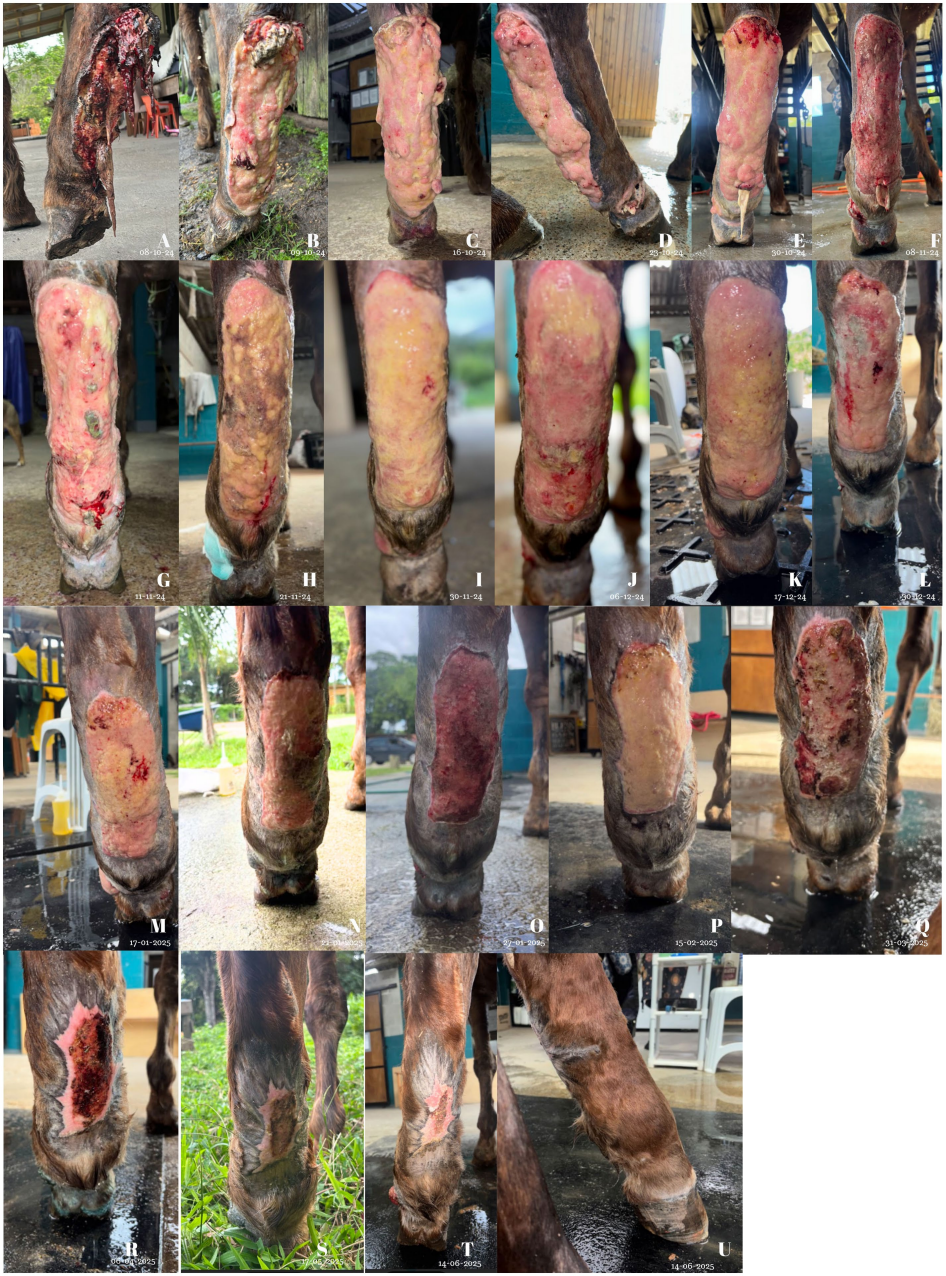
Photo documentation of the wound healing evolution **(A–U)** treated with topical solution of cannabinoids rich in THC and CBD. In the initial phase (Images **A–F**), the lesion presented as a large, circumferential ulceration with necrotic flaps, deep tissue exposure, copious purulent exudate, soft tissue edema, and exuberant verrucous granulation tissue, consistent with advanced chronic inflammation and suspected neoplastic transformation; Image F was the 1st application of copper sulfate, before the treatment with cannabinoids. During the early treatment phase (Images **G–L**), early epithelial organization and a reduction in exudation were observed, although hyperplastic granulation persisted; (Image **H**): beginning of cannabinoid treatment. Self-mutilation continued to hinder consistent epithelialization. In the mid-treatment phase (Images **M–Q**), wound borders contracted, granulationtissue matured and flattened, and signs of peripheral epithelial migration emerged. The addition of topical cannabinoid therapy appeared to correlate with improved wound tissue quality and decreased inflammation. In the final documented phase (Images **P–U**), the wound showed substantial epithelialization and fibrosis, normalization of tissue coloration.

Upon initial assessment by a local veterinarian, the horse presented with a large, chronic, ulcerative, and granulomatous lesion covering the entire left hind metatarsal region. The wound extended from the tarsometatarsal to the metatarsophalangeal joints, involving the plantar and lateral aspects of the limb (Figure 2A). Necrotic skin flaps, purulent discharge, and extensive granulation tissue were evident. The lesion’s characteristics suggested chronicity and potential involvement of deeper structures, including the palmar nerve, digital flexor tendons, and sesamoid bones. The wound had visible metatarsal medial face exposure. Two secondary circular wounds were also identified over the dorsomedial and dorsolateral aspects of the left fetlock. The horse displayed complete loss of limb function, was non-weight-bearing at rest and during movement and was assigned a lameness score of 5/5 based on the AAEP scale.

### 2.2 Diagnostics and emergency management

Blood samples were collected from the left jugular vein into EDTA and serum tubes for hematology and biochemistry. Imaging was not immediately available but was subsequently requested to assess deeper structural involvement. Mechanical debridement revealed purulent exudate, and the wound was disinfected with povidone-iodine followed by chlorhexidine. Samples from primary and secondary lesions were collected for histopathology. The site of sampling was carefully clean with chlorhexidine 2% and iodine 1%. At the margins of the lesions local anesthetic was used (1 mL of lidocaine). Samples were taken from the middle and margin of each lesion with a size of 1 cm × 1 cm and full thickness of the skin or 1 cm deep in the wound, with the use of a scalper blade n°24. Immediately fixed with Formalin 10% and stored at +4 degrees Celsius for 24 h. Following day samples were sent to the laboratory for in-house processing and further analysis with hematoxylin and eosin (H&E) (39).

Supportive care included 24 h of intravenous fluids (5% glucose in 0.9% NaCl at 3.5 L/h) and oral deworming. The horse received flunixin meglumine (1.1 mg/kg IV) and penicillin G benzathine (20,000 IU/kg IM) for 7 days. Silver sulfadiazine was applied topically, and a compressive bandage was changed daily. Corticosteroid therapy was withheld pending histopathological results, and surgical intervention was not feasible due to limited on-site resources.

### 2.3 Early disease progression and analgesic management

On the following day, clinical reassessment revealed reduced appetite, exaggerated pain expression (not loading the affected limb, reluctance to move/standup/lay down, fascial muscle contractions, high heart rate, sweating, tail flipping constantly, muscles spasms in the overload limb), and continued non-functionality of the affected limb. Due to insufficient pain control, a regimen of flunixin meglumine (2 mg/kg IM) and morphine (6 mg IM every 6 h) was initiated for two consecutive days.

After 1 week, the primary wound displayed rapidly proliferating, verrucous, and hyperplastic granulation tissue, with intermittent purulent secretion. The secondary lesions also developed similar tissue characteristics, presenting as raised, hyperplastic nodules with constricted dermal margins, forming pendulous structures. Pain control remained inconsistent and inadequate, and caregivers reported suspected sleep deprivation, likely due to the horse’s inability to lie down and rise because of pain and mechanical dysfunction. The owners reported persistent self-mutilation behavior, with the horse repeatedly biting at the wound site. This behavior was likely driven by intense pruritus and resulted in the frequent destruction of bandages, significantly increasing the number of dressing changes required per day and, consequently, the overall cost of care. Additionally, the repeated trauma to the wound caused by self-biting contributed to tissue damage and delayed healing, creating a cycle of irritation, injury, and impaired recovery.

Rescue analgesia with flunixin meglumine, morphine, and/or phenylbutazone was prescribed on an as-needed basis.

### 2.4 Surgical debridement and referral

A chemical debridement was performed using Pradovillatte® and copper sulfate. Despite this, the granulation tissue recurred aggressively within days, with continued signs of pain, anorexia, and deteriorating quality of life. Due to the location of the lesion, the progressive nature of the pathology, and financial limitations, surgical resection and electrochemotherapy were ruled out. Euthanasia was discussed as a compassionate alternative by the primary caregivers. However, before proceeding with euthanasia, the patient was referred to the *Pet Cannabis® Social Clinical Program*,^1^ for specialized equine cannabinoid-based therapy with the aim of palliating pain, managing chronic systemic inflammation and supporting welfare.

### 2.5 Diagnostic workup

#### 2.5.1 Radiograph findings

Radiographic evaluation of the left hind limb revealed extensive circumferential soft tissue mineralization along the metatarsal diaphysis, consistent with dystrophic calcification or reactive periosteal new bone formation, suggestive of chronic aggressive soft tissue pathology (Supplementary Figure 1). Articular margins of the metatarsophalangeal joint were preserved, and proximal sesamoid bones showed no lysis or fragmentation. Mild cortical irregularity and subperiosteal reaction of the caudal cortex of the third metatarsal bone were observed, compatible with early osteomyelitis or reactive inflammatory change.

#### 2.5.2 Histopathology findings

Histological evaluation revealed a dermal proliferation of spindle-shaped to stellate cells with large oval nuclei, mild anisokaryosis, and pleomorphism, arranged in interlacing nests and fascicles within a collagen-rich stroma (Supplementary Figure 2). The overlying epidermis showed parakeratotic hyperkeratosis, surface necrosis, and ulceration. Findings were consistent with a low limb equine fibroblastic sarcoid.

#### 2.5.3 Laboratorial findings at initial presentation

Initial CBC and serum biochemistry (Supplementary Figure 3) revealed moderate non-regenerative, normocytic, normochromic anemia, with neutrophilia and mild lymphopenia. Platelet count, plasma protein, liver enzymes, alkaline phosphatase, and urea were within reference ranges. AST elevation was observed and considered consistent with muscle catabolism and tissue injury.

## 3 Diagnostic assessment

Comprehensive diagnostic assessment identified a chronic neoplastic wound consistent with a fibroblastic equine sarcoid, complicated by complete suspensory tendon rupture and early bone involvement. Due to refractory pain, severe malnutrition, and poor prognosis, the case was managed under palliative care, and a cannabinoid-based protocol using oral and topical THC-rich full-spectrum cannabis oil was initiated to support comfort and pain control.

## 4 Therapeutic intervention

### 4.1 Product description

Full spectrum cannabis oil containing 100 mg/mL of THC, formulated in extra virgin olive oil as the carrier. Administered in a 1:1 ratio with a second full spectrum oil containing 100 mg/mL of CBD, also formulated in olive oil (Supplementary Material 1). Both oils were obtained through the *Santa Cannabis Associação Brasileira de Cannabis Medicinal*,^2^ a non-governmental organization facilitating access to medicinal cannabis under medical supervision. Products were labeled with cannabinoid content, batch number, and expiration date. Additionally, morphine was prescribed on demand.

### 4.2 Protocol and treatment duration

Based on clinical, imaging, and histopathological findings and poor response to conventional therapies, a long-term cannabinoid-based protocol was initiated. The target maintenance dose was 0.5 mg/kg of THC and 0.5 mg/kg of CBD administered orally every 12 h over a 10-month period. Due to the use of high-concentration full-spectrum oils (100 mg/mL), a gradual dose-escalation protocol was implemented to minimize adverse effects (soft feces, ataxia, sedation, overeating, reduction of gut movements) (29, 31).

Treatment began in November 2024 at 0.05 mg/kg of each compound (0.2 mL per oil) every 8 h in a 390-kg horse, with weekly dose escalation. Cannabinoids were doubled every 7 days, and each dose was maintained for 1 week before further increase (Table 1). Once the targeted dose (0.5 mg/kg each) was reached, administration was adjusted to twice daily.

**Table 1:** Overview of the dose protocol of cannabinoid solution.

Week	THC/CBD dose (mg/kg)	Total dose per compound (mg)	Volume per oil (mL)
Week 8 (Nov 25–Dec 1)	0.05 mg/kg	19.5 mg	0.2 mL
Week 9 (Dec 2–Dec 8)	0.10 mg/kg	39 mg	0.4 mL
Week 10 (Dec 9–Dec 15)	0.20 mg/kg	78 mg	0.8 mL
Week 11 (Dec 16–Dec 22)	0.32 mg/kg	125 mg	1.25 mL
Week 12 (Dec 23–…)	0.50 mg/kg (target)	195 mg	1.95 mL ≈ **2.0 mL**

Scalation protocol was established to avoid undesirable effects. Bold value means maximum volume used.

The combined 1:1 THC: CBD formulation was also applied topically once to twice daily to the coronary band, hoof wall, and adjacent soft tissues, either by direct massage or via gauze under occlusive bandaging. Wound care included routine cleansing with 2% chlorhexidine, continued topical cannabinoid application, and bacteriological culture with antibiogram.

### 4.3 Safety monitoring, adverse event assessment and pain monitoring

Throughout the treatment period, the patient was closely monitored every 24 h for potential adverse effects commonly associated with cannabinoid administration. The clinical parameters assessed included: changes in fecal consistency, clinical signs of colic, signs of dysphagia, cardiorespiratory abnormalities, ataxia or any neurological or cognitive alterations. Clinical signs were accessed by means of clinical examination (using a stethoscope; vital parameters, gut auscultation, general palpation, neurological examination and gait analysis), feces were accessed by means of daily direct exam.

Pain was evaluated daily using a combination of physiological and behavioral parameters, including heart rate, respiratory rate, facial muscle tension, facial expression (nostril dilation and ear position), tail movement, weight-bearing distribution between the affected and contralateral limbs, mobility (ability to stand up and lie down), willingness to move, appetite, and degree of lameness. These indicators were selected based on the equine pain ethogram (40–42) and established clinical signs associated with pain in horses (33). Although these parameters informed the evaluation, no formal composite pain scoring system was applied. Instead, the veterinary team performed a daily clinical assessment integrating all observed parameters to access pain and manage it.

## 5 Follow-up and outcomes

### 5.1 Sarcoid and wound outcome

Following initiation of systemic and topical cannabinoid therapy, the sarcoid wound showed marked clinical improvement, with progressive border regularization, reduction of granulation tissue, and formation of a smoother wound bed. Pruritus and self-mutilation resolved within 2 weeks of topical cannabinoid use. Of the additional lesions, the medial lesion fully resolved within 4 months, while the lateral lesion showed significant size reduction despite evolving into a pedunculated mass (Figure 2).

### 5.2 Joint and hoof disease outcome

After initiation of cannabinoid therapy, lameness and mobility improved transiently. Baseline lameness was grade 5/5 (AAEP) with uncontrolled pain despite conventional analgesia. Following treatment initiation (November 21, 2024), the horse regained the ability to lie down and rise, with improvement to grade 3 by December 2024 and brief trotting observed in January 2025. Intermittent deterioration occurred due to secondary complications, and from June to September 2025 lameness progressed to grade 5 despite intensified analgesia, leading to euthanasia for refractory pain (Table 2).

**Table 2:** Detailed chronological timeline summarizing the evolution of clinical findings, diagnostic procedures, and therapeutic interventions throughout the course of the disease.

Week	Event/notes	Findings/intervention
0–8	Initial evaluation by local veterinarian; Poor wound response; Refractory Pain.	Body Condition Score (BCS): 1/5. Lameness Grade: 5/5. Ulcerated, contaminated wound on left hindlimb. Poor pain control; non-responsive exuberant granulation tissue. Pain management (flunixin 1.1 mg/kg SID 7 days; morphine hydrochloride 0.2 mg/kg IM BID 5 days), (wound care + Pentabiótico 5 mL/100 kg 2 doses 5 days distant from each other), diagnostics (image exams, bloodwork, histopathology).
8	Progressive deterioration of the health state. Initiation of cannabinoid therapy (monomodal). Confirmed equine fibroblastic sarcoid (histopahotlogy).	Refractory pain. Signs of colic. Self-mutilation behavior. BCS: 1/5. Lameness: 5/5. Start of Oral and topical administration of full-spectrum THC + CBD oils.
9–20	Marked reduction in pruritus and pain. Ongoing cannabinoid therapy.	Increased appetite. Cessation of self-mutilation. Granulation tissue under control. BCS: 3/5; Lameness: improved to Grade 3/5; Horse able to lie down and stand independently;
20	Acute increase in pain - Farrier visit: abscess and keratoma identified and addressed. Wound remained moist and well-epithelialized	Lameness worsened to Grade 5/5. BCS: 4/5. THC + CBD oil dosage doubled for 48 h (0.5 to 1 mg/kg THC + 1 to 2 mg/kg CBD). Pain relief observed.
21–22	Post-keratoma intervention. Continued cannabinoid protocol. No more morphine. Wound contraction and re-epithelialization initiated. Patient able to trot short distances.	Lameness improved to Grade 3/5. Significant improvement in pain and function. BCS: 4/5
31	Sudden pain flare-up. Lameness returned to Grade 5/5. Regional limb perfusion with gentamicin (3 treatments, 72 h apart - 1 g diluted in 100 mL of sterile saline solution). Procain penicillin (30,000 iu/kg IM every 24 h for 7 days)	Radiographic imaging performed. Diagnosis: osteomyelitis. Antibiotic Therapy (Procain penicillin 30,000 iu/kg IM every 24 h for 7 days + Regional Perfusion of Gentamicin 1 g diluted in 100 mL of sterile saline solution). Nightly morphine (0.1 mg/kg IM SID 2 days) + doubled oil dosage (0.5 to 1 mg/kg THC + 1 to 2 mg/kg CBD for 10 days). After 24 h, reduction in pain. Decreased swelling of fetlock region
50–60	End-stage disease progression. Wound fully closed.	Lameness: 5/5; Anorexia. 2 mg/kg THC + 4 mg/kg CBD every 8 h + morphine 0.1 mg/kg IM every 8 h. Ataxia + Sedation + Sleepness + Peristalsis reduction Pain remained refractory.
61	Euthanasia elected by owners.	

### 5.3 Functional outcomes

Following initiation of full-spectrum cannabinoid therapy, the horse showed sustained improvement in appetite, behavior, and body condition. Appetite returned within weeks, and body condition score increased from 1/5 to 4/5 by month five (Figure 1). Behavioral distress and self-mutilation resolved. Functional improvements included independent rising, limited trotting, and increased limb use, despite a confirmed complete tendinous rupture.

### 5.4 Safety outcomes

No gastrointestinal, neurological, or cardiorespiratory adverse effects were observed during treatment. Serial hematological and biochemical analyses showed liver and renal parameters within in-house validated reference ranges (Supplementary Figure 3), with increases in erythrocytes, hemoglobin, and hematocrit from baseline. A high rescue dose administered during the final week (5 mL; 1.25 mg/kg of each oil every 8 h) resulted in transient ataxia and sedation without evidence of systemic toxicity.

## 6 Timeline

Below, overview of the timeline of the case (Table 2).

## 7 Discussion

This case highlights the effectiveness of cannabinoid therapy in managing chronic and refractory pain, particularly in a patient unresponsive to opioids, NSAIDs, and corticosteroids. Rescue doses of both cannabis oil and morphine were required only four times over the 10-month treatment period, reflecting the stability achieved through the primary cannabinoid protocol. Improvements included a progressive increase in body condition score (Figure 1), weight gain, and enhanced coat quality, indicative of improved systemic health. Topical application to the wound demonstrated strong local anti-inflammatory effects and effective control of exuberant granulation tissue. Importantly, a significant decrease in pruritus was observed, leading to complete cessation of self-mutilation within 1 week of initiating treatment. These clinical improvements contributed to a progressive enhancement in the patient’s overall quality of life, and throughout the treatment course, no adverse effects were reported during standard dosing.

Despite the benefits, cannabinoid therapy showed no observable effect on the progression of late diagnosed osteomyelitis. In the final 30 days of life, the infection proved unresponsive to antibiotic therapy, and the pain became refractory, even when managed with high doses of THC and CBD in combination with morphine. At these escalated doses, the patient developed transient adverse effects, including ataxia, sedation, and drowsiness, though without signs of systemic toxicity. Ultimately, the osteomyelitic condition deteriorated, contributing significantly to the decision for humane euthanasia.

Pain management and regulation of inflammatory response remain challenging in equine medicine. Non-Steroidal Anti-Inflammatory Drugs (NSAIDs), such as phenylbutazone have a narrow margin of safety with severe side effects associated with excessive or prolonged usage in horses including gastroduodenal ulceration, right dorsal colitis (RDC) and renal papillary necrosis (43, 44). The use of this class of drugs is common in equine practice for orthopedic disease, laminitis, colic and neurological conditions (45–49).

Cannabidiol (CBD), a non-psychoactive phytocannabinoid derived from *Cannabis sativa*, has shown promising results in equine medicine. The dose range for cannabidiol for use in horses requires further study, with a wide range of dosages from 0.07 to 2 mg/kg orally per day having shown positive results. *In-vitro* studies demonstrated that CBD at 4 μg/mL reduced the production of inflammatory cytokines (TNF-α and IFN-γ) in peripheral blood mononuclear cells from senior horses (19).

Additionally, an *in-vivo* study using CBD oil solution given at 2 mg/kg orally for 90 days to senior horses significantly decreased inflammatory cytokine expression of IFN-γ in whole blood at day 60, and IL-6 at day 60 and 90 in CBD- treated horses, when compared to controls (placebo-treated/ 15 mL of soy) (18). Aragona et al. (21) showed that a combination of cannabinoids at a dose of 0.07 mg/kg qd every 24 h for 2 weeks was efficacious in lowering the levels of pain in horses with OA. Cannabidiol has also been reported to be effective in treating equine mechanical allodynia (20) and stereotypic behavior (29). Oral administration of cannabinoids appears to be well tolerated by horses in a wide range of doses (21, 50–53).

For the authors knowledge, there is no recent clinical study of the medical use of oral THC in horses. This case puts in evidence the clinical benefit of oral THC rich compound solution for horses and its safety in a long-term scalation dose protocol. The undesirable effects present were possibly related to THC rather than CBD administration (2 mg/kg THC + 4 mg/kg CBD). The clinical signs were ataxia, sedation, sleepiness and reduction of gut motility; not related to toxicity or shock. The presents find aligns with studies using compounds with THC in other species (54–57).

The role of cannabinoids in wound healing is an emerging area of investigation. These compounds are known to modulate inflammatory cell infiltration (58) and cellular proliferation (59, 60) —key processes involved in both physiological and pathological wound repair. In humans and mice fibroblasts specifically, CBD has been shown to influence the activity of matrix metalloproteinases (MMPs) (61–63), enzymes critical for extracellular matrix turnover. This is particularly relevant in the context of equine sarcoids, where MMPs are implicated in tumor progression, local invasiveness, and potential malignancy (64–66).

It is hypothesized that cannabinoids may exert dual therapeutic action on the wound site: not only by promoting wound healing through anti-inflammatory, proliferative, and apoptotic mechanisms, but also by downregulating MMP activity, thus potentially inhibiting sarcoid progression. In other tumor models, such as human breast cancer (38, 67), cannabinoids have demonstrated the ability to induce cancer cell apoptosis and interfere with aromatase expression, further supporting their antineoplastic potential (38, 68). A recent paper evaluated the response of equine sarcoid cells to CBD *in vitro*, focusing on viability, invasiveness, and matrix remodeling (69). Treatment with CBD affected cell viability, cytotoxicity, and apoptosis. At 48 h, apoptosis (measured as caspase 3/7 activity) reached 49.5% and further increased to 75% at 72 h. Marked cytotoxicity (>96%) and decreased viability were observed at 72 h. Cannabidiol also significantly decreased MMP-1 concentration by 48.9% at 24 h and MMP-2 concentration after 6 h (69).

In this case, the combined anti-inflammatory and citomodulatory properties of the full-spectrum cannabinoid therapy appear to have contributed to effective granulation tissue control, enhanced re-epithelialization, and stable wound healing, all without evidence of neoplastic growth. Although cannabinoids modulate key processes involved in wound repair—such as inflammation (70), fibroblast proliferation (69), and matrix metalloproteinase (MMP) activity (69)—the relatively long period required to observe clinical resolution in the present case (approximately 8 months) may also be related to the pharmaceutical formulation used. The cannabinoids were administered in a conventional oil-based formulation without the use of advanced drug-delivery technologies. Oral oil preparations of lipophilic molecules such as cannabinoids are known to present variable gastrointestinal absorption and relatively low bioavailability due to extensive first-pass hepatic metabolism (30, 71, 72), which may reduce the proportion of the active compound reaching systemic circulation and target tissues. Consequently, sustained administration over extended periods may be required before clinically significant effects become evident.

Similarly, although topical application was employed directly at the wound site, conventional oil-based preparations may also present limited transdermal penetration when compared with advanced delivery systems. The diffusion of cannabinoids through the skin barrier is influenced by multiple factors, including formulation characteristics, molecular solubility, and carrier systems (73). Advanced pharmaceutical technologies such as nanoencapsulation or liposomal formulations have been developed to enhance drug penetration across biological membranes and improve tissue targeting (74). These systems can increase local absorption, improve bioavailability, and reduce metabolic degradation, thereby enhancing therapeutic exposure at the target site (74–77). In human medicine, particularly in oncology, liposomal and nanoparticle-based delivery platforms are widely used to optimize the delivery and efficacy of chemotherapeutic agents (67, 78). Emerging evidence suggests that similar strategies may also enhance the therapeutic potential of cannabinoids (79–81).

Regarding appetite stimulation, this effect is well established in human clinical trials. In patients with systemic sclerosis associated with anorexia and malnutrition, treatment with THC and CBD in a 1:1 ratio resulted in improvements in appetite, satisfaction with eating, ability to eat more, body weight and daily calorie intake (82). In addition, THC has been widely used in oncologic patients and, beyond its orexigenic effects, has demonstrated an ability to attenuate weight loss in individuals with advanced malignant tumors (83). There is a possible involvement of CB1 receptors located in the hypothalamus, where food intake is regulated, as well as the mesolimbic reward system, which plays a role in the motivation and reward aspects of feeding (84). There is a lack of information regarding appetite stimulation in horses. Cunha et al. (29) described an increase in appetite 1 hour after isolated CBD administration for the treatment of crib-biting. After 1 week of treatment, the horse showed a sustained enhancement of appetite, and by the end of 30 days, an improvement in body condition score (BCS) was observed.

In this case, several practical limitations and inherent risks must be acknowledged. The range of therapeutic and procedural options was constrained by a combination of financial limitations on the part of the owners and the geographical location of the case, which affected the availability of advanced veterinary interventions. In an ideal scenario, the patient would have benefited from surgical debridement, extensive wound flushing, surgical antibiotic protocols, and possibly the placement of drainage systems to manage the infection more aggressively. Another limitation of this study is the absence of a validated and standardized pain scoring system. Pain assessment was performed daily by the veterinary team through clinical observation of behavioral and physiological parameters consistent with the equine pain ethogram (42) and known pain-related behaviors (85). However, because the evaluation relied on qualitative clinical judgment rather than an validated composite pain scale, a degree of subjectivity cannot be excluded and comparisons with studies using standardized pain assessment tools may be limited.

Although the histopathological diagnosis was consistent with an equine fibroblastic sarcoid, it should be acknowledged that in extensive and highly reactive lesions such as the one described in this case, histopathological differentiation between exuberant granulation tissue and sarcoid tissue can be challenging and is not always definitive (86). In severe chronic wounds, marked inflammation, fibrosis, and reactive tissue proliferation may obscure classical histological features, potentially limiting diagnostic accuracy (87). Additionally, although copper sulfate was applied locally during wound management, the authors recognize that this approach is not recommended in contemporary equine wound care due to its potential to cause tissue irritation and delayed healing (88, 89). Its use in this case highlights the practical challenges frequently encountered in long-term cases, where owner-driven treatments and variable compliance with veterinary recommendations may influence wound management strategies.

The necessity to rely on a palliative, multimodal approach—involving cannabinoids alongside other analgesics—was in part due to these constraints. While this approach succeeded in improving quality of life significantly, it is important to recognize that the outcome of euthanasia reflects the severity of the underlying condition and the challenges inherent in managing advanced neoplastic and infectious processes in a resource-limited setting. In discussing the limitations and risks of this case, it is important to emphasize that this is a single clinical case report. The patient required intermittent doubling of the cannabinoid dose and the addition of morphine during certain acute phases, highlighting the severity of the pain and the clinical challenge of managing such a complex case. This underscores the necessity of a multimodal approach, integrating multiple therapeutic agents to achieve palliation.

From the authors’ perspective and clinical experience with prescribing cannabinoids in horses, both THC and CBD may represent a potential shift in how equine veterinarians manage pain and, consequently, the patient. With the use of cannabinoids, pain appears to be markedly attenuated, within the limits imposed by dosage and safety considerations. While this represents a positive step toward improving comfort and appropriately addressing pain in equine patients, it may also introduce a clinical challenge: the need for substantially intensified follow-up and monitoring. When pain is effectively managed, certain clinical signs may be masked or reduced, potentially delaying the recognition of disease progression or complications. As a result, closer and more frequent clinical evaluations become essential. This consideration does not disqualify cannabinoids as a therapeutic tool; on the contrary, their use may significantly enhance comfort and overall well-being in suffering equine patients, provided that adequate clinical surveillance is maintained.

It is also crucial to note that although the outcome was euthanasia, the primary goal of palliative care was achieved, as the patient’s quality of life significantly improved during the treatment period. The use of full-spectrum oils containing 100 mg/mL of both THC and CBD, along with other cannabinoids, likely contributed to the safety and therapeutic balance of the protocol. The entourage effect of full-spectrum cannabis may enhance analgesic and anti-inflammatory benefits while minimizing adverse effects within a therapeutic window (90, 91).

Following treatment initiation, the owner observed visible improvement in wound appearance, reduced exudate, and healthier tissue margins, accompanied by decreased discomfort and distress. Ongoing communication with the veterinary team, supported by regular photographic and video updates, guided therapeutic adjustments and reinforced collaborative decision-making. The owner identified the cannabis-based therapy as the first intervention to provide sustained clinical improvement and stability, reporting a meaningful enhancement in the horse’s quality of life despite eventual disease progression and euthanasia.

## 8 Clinical relevance and conclusion

This case suggests that long-term administration of THC-rich and CBD-rich cannabis oils may be a useful adjunct for palliative management in horses with chronic, refractory conditions. Gradual dose escalation was clinically well tolerated, with only transient, dose-dependent adverse effects observed at high rescue doses. Cannabinoid therapy was associated with improved comfort, mobility, and quality of life, supporting its potential role within multimodal palliative care. Further controlled studies are warranted to define optimal dosing and clinical indications.

## Data Availability

The original contributions presented in the study are included in the article/Supplementary material, further inquiries can be directed to the corresponding author.

## Footnotes

1
https://abpetcannabis.com.br/

2
https://santacannabis.com.br/

## Supplementary material

SUPPLEMENTARY MATERIAL 1 Cannabinoid oil analysis.

SUPPLEMENTARY MATERIAL 2 Radiographic (Supplementary Figure 1) and histopathologic diagnosis (Supplementary Figure 2); Longitudinal comparison of hemogram and biochemical parameters (Supplementary Figure 3).

## References

[1] Silver RJ. The endocannabinoid system of animals. Animals (Basel). (2019) 9:686. doi: 10.3390/ani9090686, 31527410 PMC6770351

[2] Yang HM Kim J Kim BK Seo HJ Kim JY Lee JE . Resistin regulates inflammation and insulin resistance in humans via the endocannabinoid system. Research (Wash D C). (2024) 6:326. doi: 10.34133/research.0326, 39050819 PMC11267475

[3] Dörnyei G Vass Z Juhász CB Nádasy GL Hunyady L Szekeres M. Role of the endocannabinoid system in metabolic control processes and in the pathogenesis of metabolic syndrome: an update. Biomedicine. (2023) 11:306. doi: 10.3390/biomedicines11020306, 36830844 PMC9952954

[4] Sládek M Houdek P Sumová A. Circadian profiling reveals distinct regulation of endocannabinoid system in the rat plasma, liver and adrenal glands by light-dark and feeding cycles. Biochim Biophys Acta Mol Cell Biol Lipids. (2019) 1864:158533. doi: 10.1016/j.bbalip.2019.158533, 31676438

[5] Di Marzo V Ligresti A Cristino L. The endocannabinoid system as a link between homoeostatic and hedonic pathways involved in energy balance regulation. Int J Obes. (2009) 33:S18–24. doi: 10.1038/ijo.2009.67, 19528974

[6] Di Marzo V Piscitelli F. The endocannabinoid system and its modulation by Phytocannabinoids. Neurotherapeutics. (2015) 12:692–8. doi: 10.1007/s13311-015-0374-6, 26271952 PMC4604172

[7] Zamith Cunha R Salamanca G Mille F Delprete C Franciosi C Piva G . Endocannabinoid system receptors at the hip and stifle joints of middle-aged dogs: a novel target for the therapeutic use of *Cannabis sativa* extract in canine Arthropathies. Animals. (2023) 13:2833. doi: 10.3390/ani13182833, 37760233 PMC10525782

[8] Polidoro G Galiazzo G Giancola F Papadimitriou S Kouki M Sabattini S . Expression of cannabinoid and cannabinoid-related receptors in the oral mucosa of healthy cats and cats with chronic gingivostomatitis. J Feline Med Surg. (2021) 23:679–91. doi: 10.1177/1098612X20970510, 33174485 PMC10812186

[9] Bombardi C Salamanca G Tagliavia C Grandis A Zamith Cunha R Gramenzi A . Cannabinoid receptors in the horse lateral nucleus of the amygdala: a potential target for ameliorating pain perception, stress and anxiety in horses. Int J Mol Sci. (2025) 26:7613. doi: 10.3390/ijms26157613 40806746 PMC12347058

[10] Zamith Cunha R Gobbo F Morini M Zannoni A Mainardi C D’arpe L . Distribution of endocannabinoid system receptors in the equine hoof: dysregulation as a potential therapeutic target for laminitis. Histochem Cell Biol. (2025) 163:71. doi: 10.1007/s00418-025-02397-y, 40593311

[11] Chiocchetti R Salamanca G De Silva M Gobbo F Aspidi F Cunha RZ . Cannabinoid receptors in the inflammatory cells of canine atopic dermatitis. Front Vet Sci. (2022) 9:987132. doi: 10.3389/fvets.2022.987132, 36187821 PMC9521433

[12] Petrie GN Nastase AS Aukema RJ Hill MN. Endocannabinoids, cannabinoids and the regulation of anxiety. Neuropharmacology. (2021) 195:108626. doi: 10.1016/j.neuropharm.2021.108626, 34116110

[13] Corsetti S Borruso S Malandrucco L Spallucci V Maragliano L Perino R . *Cannabis sativa* L. may reduce aggressive behaviour towards humans in shelter dogs. Sci Rep. (2021) 11:2773. doi: 10.1038/s41598-021-82439-2, 33531559 PMC7854708

[14] Conrow KD Haney RS Malek-Ahmadi MH Albright JD Kaplan BLF Snyder-Mackler N . Demographic features, health status, and behavioral changes associated with cannabidiol use in the dog aging project. Front Vet Sci. (2025) 12:1666663. doi: 10.3389/fvets.2025.1666663, 41394909 PMC12698446

[15] Calheiros LGR De M Pedro G Oliveira Da Silva T Amorim RM CEF A . In vitro antitumor effect of oils rich in CBD and THC cannabis extract in canine prostate carcinoma cell lines. Vet Sci. (2024) 10:501. doi: 10.3390/vetsci11100501 PMC11512242 39453093

[16] Omer S Mansour M Pondugula SR Dhanasekaran M Matz B Khan O . Cytotoxicity of cannabinoids in combination with traditional lymphoma chemotherapeutic drugs against non-Hodgkin’s lymphoma. Biomedicine. (2025) 14:3. doi: 10.3390/biomedicines14010003, 41595541 PMC12837863

[17] Wang B Li D Fiselier A Kovalchuk I Kovalchuk O. High-CBD cannabis extracts inhibit the expression of proinflammatory factors via miRNA-mediated silencing in human small intestinal epithelial cells. Heliyon. (2023) 9:e18817. doi: 10.1016/j.heliyon.2023.e18817, 37664748 PMC10468390

[18] Turner S Knych HK Adams AA. The effects of cannabidiol on immune function and health parameters in senior horses. Vet Immunol Immunopathol. (2023) 257:110549. doi: 10.1016/j.vetimm.2023.110549, 36682327

[19] Turner S Barker VD Adams AA. Effects of Cannabidiol on the in vitro lymphocyte pro-inflammatory cytokine production of senior horses. J Equine Vet Sci. (2021) 103:103668. doi: 10.1016/j.jevs.2021.103668, 34281647

[20] Ellis KL Contino EK. Treatment using cannabidiol in a horse with mechanical allodynia. Equine Vet Educ. (2021) 33:e79–82. doi: 10.1111/eve.13168, 40046247

[21] Aragona F Tabbì M Gugliandolo E Giannetto C D’Angelo F Fazio F . Role of cannabidiolic acid or the combination of cannabigerol/cannabidiol in pain modulation and welfare improvement in horses with chronic osteoarthritis. Front Vet Sci. (2024) 11:1496473. doi: 10.3389/fvets.2024.1496473, 39720409 PMC11668182

[22] Carroll AT Reed RA Berghaus LJ McNabney D Knych HK. Oral cannabidiol increases thermal threshold in horses without physiologic adverse effects. Am J Vet Res. (2025) 86:ajvr.25.05.018. doi: 10.2460/ajvr.25.05.0185, 40854532

[23] Zylla DM Eklund J Gilmore G Gavenda A Guggisberg J VazquezBenitez G . A randomized trial of medical cannabis in patients with stage IV cancers to assess feasibility, dose requirements, impact on pain and opioid use, safety, and overall patient satisfaction. Support Care Cancer. (2021) 29:7471–8. doi: 10.1007/s00520-021-06301-x, 34085149

[24] Chhabra M Ben-Eltriki M Paul A Lê M Herbert A Oberoi S . Cannabinoids for symptom management in children with cancer: a systematic review and meta-analysis. Cancer. (2023) 129:3656–70. doi: 10.1002/cncr.34920, 37635461

[25] Makary P Parmar JR Mims N Khanfar NM Freeman RA. Patient counseling guidelines for the use of cannabis for the treatment of chemotherapy-induced nausea/vomiting and chronic pain. J Pain Palliat Care Pharmacother. (2018) 32:216–25. doi: 10.1080/15360288.2019.1598531, 31070496

[26] Valente AC Lopes LPN Matheus ME. Medical cannabis use in oncology and associated outcomes: a scoping review. J Oncol Pharm Pract. (2024) 30:737–51. doi: 10.1177/10781552241239006, 38477532

[27] Brown D Watson M Schloss J. Pharmacological evidence of medicinal cannabis in oncology: a systematic review. Support Care Cancer. (2019) 27:3195–207. doi: 10.1007/s00520-019-04774-5, 31062109

[28] Haney M Choo TH Tiersten A Levin FR Grassetti A DeSilva N . Oral cannabis for Taxane-induced neuropathy: a pilot randomized placebo-controlled study. Cannabis Cannabinoid Res. (2025) 10:631–9. doi: 10.1089/can.2025.0028, 40611810

[29] Cunha RZ Felisardo LL Salamanca G Marchioni GG Neto OI Chiocchetti R. The use of cannabidiol as a novel treatment for oral stereotypic behaviour (crib-biting) in a horse. Vet Anim Sci. (2023) 19:100289. doi: 10.1016/j.vas.2023.100289, 36824298 PMC9941357

[30] Turner SE Knych HK Adams AA. Pharmacokinetics of cannabidiol in a randomized crossover trial in senior horses. Am J Vet Res. (2022) 83:ajvr.22.02.0028. doi: 10.2460/ajvr.22.02.0028, 35895770

[31] Fitzgerald AH Magnin G Pace E Bischoff K Pinn-Woodcock T Vin R . Marijuana toxicosis in 2 donkeys. J Vet Diagn Invest. (2022) 34:539–42. doi: 10.1177/10406387211064269, 35037522 PMC9254068

[32] Haspeslagh M Gerber V Knottenbelt DC Schüpbach G Martens A Koch C. The clinical diagnosis of equine sarcoids—part 2: assessment of case features typical of equine sarcoids and validation of a diagnostic protocol to guide equine clinicians in the diagnosis of equine sarcoids. Vet J. (2018) 240:14–8. doi: 10.1016/j.tvjl.2018.08.010, 30268326

[33] Guedes A. Pain Management in Horses. Vet Clin N Am Equine Pract. (2017) 33:181–211. doi: 10.1016/j.cveq.2016.11.006, 28325179

[34] Sermsaksasithorn P Nopsopon T Samuthpongtorn C Chotirosniramit K Pongpirul K. Cannabis and cannabinoids in dermatology: protocol for a systematic review and meta-analysis of quantitative outcomes. BMJ Open. (2023) 13:e075007. doi: 10.1136/bmjopen-2023-075007, 37699631 PMC10503344

[35] Klein M Quadros De Bortolli J Guimarães FS Salum FG Cherubini K MAZ De Figueiredo. Effects of cannabidiol, a *Cannabis sativa* constituent, on oral wound healing process in rats: clinical and histological evaluation. Phytother Res (2018);32:2275–2281. doi:10.1002/ptr.6165 30088305

[36] Maida V Shi RB Fazzari FGT Zomparelli L. Topical cannabis-based medicines – a novel paradigm and treatment for non-uremic calciphylaxis leg ulcers: an open label trial. Int Wound J. (2020) 17:1508–16. doi: 10.1111/iwj.13484, 32875692 PMC7540661

[37] Heath DM Koslosky EJ Bartush KC Hogue GD. Marijuana in orthopaedics: effects on bone health, wound-healing, surgical complications, and pain management. JBJS Rev. (2022) 10:e21.00184. doi: 10.2106/JBJS.RVW.21.00184, 35180183

[38] Fleege NMG Miller EA Kidwell KM Zacharias ZR Houtman J Scheu K . Pilot study of Cannabidiol for treatment of aromatase inhibitor-associated musculoskeletal symptoms in breast cancer. Cancer Med. (2025) 14:e71117. doi: 10.1002/cam4.71117, 40751295 PMC12316815

[39] Martens A De Moor A Demeulemeester J Ducatelle R. Histopathological characteristics of five clinical types of equine sarcoid. Res Vet Sci. (2000) 69:295–300. doi: 10.1053/rvsc.2000.0432, 11124103

[40] Dyson S Pollard D. Application of the ridden horse pain Ethogram to horses competing at the Hickstead-Rotterdam grand prix challenge and the British dressage grand prix National Championship 2020 and comparison with world cup grand prix competitions. Animals. (2021) 11:1820. doi: 10.3390/ani11061820, 34207251 PMC8235099

[41] Dyson S Pollard D. Application of a ridden horse pain Ethogram and its relationship with gait in a convenience sample of 60 riding horses. Animals. (2020) 10:1044. doi: 10.3390/ani10061044, 32560486 PMC7341225

[42] Dyson S Pollard D. Application of the ridden horse pain Ethogram to elite dressage horses competing in world cup grand prix competitions. Animals. (2021) 11:1187. doi: 10.3390/ani11051187, 33919208 PMC8143096

[43] Flood J Stewart AJ. Non-steroidal anti-inflammatory drugs and associated toxicities in horses. Animals. (2022) 12:2939. doi: 10.3390/ani12212939, 36359062 PMC9655344

[44] Ahmadnejad M Jalilzadeh-Amin G Sykes BW. Prophylactic effects of *Glycyrrhiza glabra* root extract on phenylbutazone-induced equine glandular gastric disease (EGGD). J Equine Vet Sci. (2022) 118:104088. doi: 10.1016/j.jevs.2022.104088, 35908599

[45] Jacobs CC Schnabel LV McIlwraith CW Blikslager AT. Non-steroidal anti-inflammatory drugs in equine orthopaedics. Equine Vet J. (2022) 54:636–48. doi: 10.1111/evj.13561, 35076950 PMC9304133

[46] Sheats MK. A comparative review of equine SIRS, sepsis, and neutrophils. Front Vet Sci. (2019) 6:69. doi: 10.3389/fvets.2019.00069, 30931316 PMC6424004

[47] Bell T Kyriazopoulou P Mowbray C Murphy BA. Equine headshaking syndrome: triggers, seasonality, and treatment efficacy in Australia. Animals. (2024) 14:875. doi: 10.3390/ani14060875, 38539973 PMC10967644

[48] Roy MF. Sepsis in adults and foals. Vet Clin N Am Equine Pract. (2004) 20:41–61. doi: 10.1016/j.cveq.2003.12.005, 15062458

[49] Parks A O’Grady SE. Chronic laminitis: current treatment strategies. Vet Clin N Am Equine Pract. (2003) 19:393–416. doi: 10.1016/S0749-0739(03)00019-1, 14575166

[50] Yocom AF O’Fallon ES Gustafson DL Contino EK. Pharmacokinetics, safety, and synovial fluid concentrations of single- and multiple-dose Oral administration of 1 and 3 mg/kg Cannabidiol in horses. J Equine Vet Sci. (2022) 113:103933. doi: 10.1016/j.jevs.2022.103933, 35307550

[51] Trevisiol S Popot MA Garcia P Boyer S Caroff M Drif L . In vivo comparative study of hemp straw exposure and cannabidiol oil administration in horse urine. Drug Test Anal. (2024) 17:805–11. doi: 10.1002/dta.3783, 39118356

[52] Thomson ACS McCarrel TM Zakharov A Gomez B Lyubimov A Schwark WS . Pharmacokinetics and tolerability of single-dose enteral cannabidiol and cannabidiolic acid rich hemp in horses (*Equus caballus*). Front Vet Sci. (2024) 11:1356463. doi: 10.3389/fvets.2024.1356463, 38681854 PMC11047043

[53] Interlandi C Tabbì M Di Pietro S D’Angelo F Costa GL Arfuso F . Improved quality of life and pain relief in mature horses with osteoarthritis after oral transmucosal cannabidiol oil administration as part of an analgesic regimen. Front Vet Sci. (2024) 11:1341396. doi: 10.3389/fvets.2024.1341396, 38379920 PMC10876772

[54] Lyons C McEwan K Munn-Patterson M Vuong S Alcorn J Chicoine A. Pharmacokinetic of two oral doses of a 1:20 THC:CBD cannabis herbal extract in cats. Front Vet Sci. (2024) 11:1352495. doi: 10.3389/fvets.2024.1352495, 38585296 PMC10996858

[55] Szkudlarek HJ Rodríguez-Ruiz M Hudson R De Felice M Jung T Rushlow WJ . THC and CBD produce divergent effects on perception and panic behaviours via distinct cortical molecular pathways. Prog Neuro-Psychopharmacol Biol Psychiatry. (2021) 104:110029. doi: 10.1016/j.pnpbp.2020.110029, 32623021

[56] Anand U Oldfield C Pacchetti B Anand P Sodergren MH. Dose-related inhibition of capsaicin responses by cannabinoids CBG, CBD, THC and their combination in cultured sensory neurons. J Pain Res. (2021) 14:3603–14. doi: 10.2147/JPR.S336773, 34853533 PMC8627890

[57] da Silva MES Christianetti B Amazonas E Pereira ML. Case report: cannabinoid therapy for discoid lupus erythematosus in a dog. Front Vet Sci. (2024) 11:1309167. doi: 10.3389/fvets.2024.1309167, 38406630 PMC10884172

[58] Shrestha C Yoo EH Deshar B Hwang M Kang S Bin BH . Cannabidiol as a therapeutic agent for rosacea through simultaneous inhibition of multiple inflammatory pathways. BMB Rep. (2025) 58:357–63. doi: 10.5483/BMBRep.2024-0115, 40754776 PMC12402694

[59] Zhao Y Zhang Y Liu H Qiao X Li D Zhu Y . The activation of CB2 enhances bone remodeling in periodontitis. BMC Oral Health. (2025) 25:788. doi: 10.1186/s12903-025-06101-3, 40413460 PMC12102886

[60] Guzmán M Sánchez C Galve-Roperh I. Cannabinoids and cell fate. Pharmacol Ther. (2002) 95:175–84. doi: 10.1016/S0163-7258(02)00256-5, 12182964

[61] Ramer R Hinz B. Cyclooxygenase-2 and tissue inhibitor of matrix metalloproteinases-1 confer the antimigratory effect of cannabinoids on human trabecular meshwork cells. Biochem Pharmacol. (2010) 80:846–57. doi: 10.1016/j.bcp.2010.05.010, 20488167

[62] Ramer R Hinz B. Inhibition of cancer cell invasion by cannabinoids via increased expression of tissue inhibitor of matrix Metalloproteinases-1. JNCI J Natl Cancer Inst. (2008) 100:59–69. doi: 10.1093/jnci/djm268, 18159069

[63] Ramer R Fischer S Haustein M Manda K Hinz B. Cannabinoids inhibit angiogenic capacities of endothelial cells via release of tissue inhibitor of matrix metalloproteinases-1 from lung cancer cells. Biochem Pharmacol. (2014) 91:202–16. doi: 10.1016/j.bcp.2014.06.017, 24976505

[64] Mosseri S Hetzel U Hahn S Michaloupoulou E Sallabank HC Knottenbelt DC . Equine sarcoid: in situ demonstration of matrix metalloproteinase expression. Vet J. (2014) 202:279–85. doi: 10.1016/j.tvjl.2014.07.026, 25439440 PMC7128672

[65] Podstawski P Ropka-Molik K Semik-Gurgul E Samiec M Skrzyszowska M Podstawski Z . Assessment of BPV-1 mediated matrix metalloproteinase genes deregulation in the in vivo and in vitro models designed to explore molecular nature of equine Sarcoids. Cells. (2022) 11:1268. doi: 10.3390/cells11081268, 35455948 PMC9025493

[66] Yuan Z Gobeil PAM Campo MS Nasir L. Equine sarcoid fibroblasts over-express matrix metalloproteinases and are invasive. Virology. (2010) 396:143–51. doi: 10.1016/j.virol.2009.10.010, 19896685

[67] Yazdan M Naghib SM Moepubi MR. Liposomal Nano-based drug delivery Systems for Breast Cancer Therapy: recent advances and progresses. Anti Cancer Agents Med Chem. (2024) 24:896–915. doi: 10.2174/0118715206293653240322041047, 38529608

[68] McAllister SD Abood ME Califano J Guzmán M. Cannabinoid cancer biology and prevention. J Natl Cancer Inst Monogr. (2021) 2021:99–106. doi: 10.1093/jncimonographs/lgab008, 34850900

[69] Semik-Gurgul E Ocłoń E Zubel-Łojek J Pędziwiatr R Pawlina-Tyszko K. Cannabidiol-induced cellular and matrix-associated responses in primary equine sarcoid cells. J Vet Intern Med. (2026) 40:aalaf015. doi: 10.1093/jvimsj/aalaf015, 41742517 PMC12881954

[70] Tóth KF Ádám D Bíró T Oláh A. Cannabinoid signaling in the skin: therapeutic potential of the “c(ut)annabinoid” system. Molecules. (2019) 24:918. doi: 10.3390/molecules24050918, 30845666 PMC6429381

[71] Draeger AL. Cannabidiol in the horse: pharmacokinetics and effects of a Cannabidiol in the horse: pharmacokinetics and effects of a pelleted supplement on reactivity and movement pelleted supplement on reactivity and movement [internet]. (2020). Report Available online at: https://digitalcommons.murraystate.edu/etd (Accessed December 15, 2025).

[72] Draeger AL Hoffman LK Godwin PR Davis AJ Porr SA Draeger AL . Pharmacokinetics of a single feeding of pelleted Cannabidiol in horses. Steeplechase: An ORCA Student Journal. (2021) 4:1.

[73] Sharma VK Sarwa KK Mazumder B. Fluidity enhancement: a critical factor for performance of liposomal transdermal drug delivery system. J Liposome Res. (2014) 24:83–9. doi: 10.3109/08982104.2013.847956, 24160895

[74] Jeon SO Hwang HJ Oh DH Seo JE Chun KH Hong SM . Enhanced percutaneous delivery of recombinant human epidermal growth factor employing nano-liposome system. J Microencapsul. (2012) 29:234–41. doi: 10.3109/02652048.2011.646327, 22214321

[75] Tenchov R Bird R Curtze AE Zhou Q. Lipid nanoparticles─from liposomes to mRNA vaccine delivery, a landscape of research diversity and advancement. ACS Nano. (2021) 15:16982–7015. doi: 10.1021/acsnano.1c04996, 34181394

[76] Kim HR Cho HB Lee S Park JI Kim HJ Park KH. Fusogenic liposomes encapsulating mitochondria as a promising delivery system for osteoarthritis therapy. Biomaterials. (2023) 302:122350. doi: 10.1016/j.biomaterials.2023.122350, 37864947

[77] Yndart Arias A Vashist A Vadell K Lakshmana MK Liuzzi JP. Liposomal-Cannabidiol Nanoformulation to suppress HIV replication and reduce oxidative stress in infected microglia. ACS Biomater Sci Eng. (2025) 11:7536–53. doi: 10.1021/acsbiomaterials.5c01218, 41222925 PMC12965768

[78] Kapoor D Sharma S Verma K Bisht A Sharma M Singhai NJ . Quality-by-design-based engineered liposomal Nanomedicines to treat cancer: an in-depth analysis. Nanomedicine. (2022) 17:1173–89. doi: 10.2217/nnm-2022-0069, 36178357

[79] Shilo-Benjamini Y Cern A Zilbersheid D Hod A Lavy E Barasch D . A case report of subcutaneously injected liposomal Cannabidiol formulation used as a compassion therapy for pain management in a dog. Front Vet Sci. (2022) 9:892306. doi: 10.3389/fvets.2022.892306, 35573415 PMC9097221

[80] Franzè S Angelo L Casiraghi A Minghetti P Cilurzo F. Design of Liposomal Lidocaine/Cannabidiol fixed combinations for local neuropathic pain treatment. Pharmaceutics. (2022) 14:1915. doi: 10.3390/pharmaceutics14091915, 36145663 PMC9504077

[81] Sato H Watanabe K Yagi R Yagi A Rikimura S Shimizu Y . Comparative study on Cannabidiol-loaded solubilizing Systems for Improvement of Oral bioavailability: liposome and Cyclodextrin-based formulations. Biopharm Drug Dispos. (2025) 47:3–11. doi: 10.1002/bdd.70019, 41454837

[82] Pisprasert V Sripanichkulchai B Khannongpho T Jumnainsong A Mahakkanukrauh A Suwannaroj S . Efficacy of cannabis oil on appetite and quality of life in systemic sclerosis patients: a randomized placebo-controlled trial. J Cannabis Res. (2025) 7:82. doi: 10.1186/s42238-025-00342-3, 41137182 PMC12553150

[83] Ben AM. Cannabinoids in medicine: a review of their therapeutic potential. J Ethnopharmacol. (2006) 105:1–25. doi: 10.1016/j.jep.2006.02.001, 16540272

[84] Abrams D Guzman M. Cannabis in cancer care. Clin Pharmacol Ther. (2015) 97:575–86. doi: 10.1002/cpt.108, 25777363

[85] Ask K Rhodin M Tamminen LM Hernlund E Haubro AP. Identification of body behaviors and facial expressions associated with induced orthopedic pain in four equine pain scales. Animals. (2020) 10:2155. doi: 10.3390/ani10112155, 33228117 PMC7699379

[86] Tana C Donatiello I Caputo A Tana M Naccarelli T Mantini C . Clinical features, histopathology and differential diagnosis of sarcoidosis. Cells. (2021) 11:59. doi: 10.3390/cells11010059, 35011621 PMC8750978

[87] Knottenbelt DC Schumacher J Toth F. "Sarcoid transformation at wound sites". In: Theoret C Schumacher J, editors. Equine Wound Management. Ames, Iowa, USA: Wiley (2016). p. 490–507.

[88] Ribeiro G Carvalho L Borges J Prazeres J. The best protocol to treat equine skin wounds by second intention healing: a scoping review of the literature. Animals (Basel). (2024) 14:1500. doi: 10.3390/ani14101500, 38791717 PMC11117370

[89] Kluger N Lain E Frasson N Doat G Stennevin A Bianchi P. The multifaceted properties of copper and zinc in skin healing. Dermatol Ther (Heidelb). (2026) 16:143–54. doi: 10.1007/s13555-025-01575-z, 41205051 PMC12873029

[90] André R Gomes AP Pereira-Leite C Marques-da-Costa A Monteiro Rodrigues L Sassano M . The entourage effect in cannabis medicinal products: a comprehensive review. Pharmaceuticals. (2024) 17:1543. doi: 10.3390/ph17111543, 39598452 PMC11870048

[91] Christensen C Rose M Cornett C Allesø M. Decoding the postulated entourage effect of medicinal cannabis: what it is and what it isn’t. Biomedicine. (2023) 11:2323. doi: 10.3390/biomedicines11082323, 37626819 PMC10452568

