# Supplementary material for: Case Report: Oral and topical chronic administration of THC-rich and CBD-rich cannabis oil as palliative care in a rescued horse with open wound, sarcoid and chronic pain
Source: Front Vet Sci. 2026 Jun 2;13:1794084. doi: 10.3389/fvets.2026.1794084 (PMC13269266; doi:10.3389/fvets.2026.1794084)
Supplement: SUPPLEMENTARY MATERIAL 1 — Cannabinoid oil analysis. [file Data_Sheet_1.pdf]

CLIENTE: Associação Brasileira de Cannabis Medinal

ORÇAMENTO/PROTOCOLO: 2426.25

ENDEREÇO: Rua General Liberato Bittencourt, 1885 – Sala 504

AMOSTRA: Solução oleosa de Cannabis

LOTE: THC100001

DATA DE FABRICAÇÃO: 16/07/2025

DATA DE VALIDADE: 16/07/2026

AMOSTRAGEM: Realizada pelo cliente

DATA DE RECEBIMENTO: 04/08/2025

REALIZAÇÃO DOS ENSAIOS: 09/09/2025

EMISSÃO DOS RESULTADOS: 18/09/2025

RESULTADOS

Ensaio	Especificação do Cliente	Resultado	LQ	Método
Determinação do Teor de Canabinoides por HPLC[1]	Canabidiol (CBD + CBDA): Não informado<br><br>Tetrahidrocanabidiol (THC + THCA): 9% a 11% p/V (10% ± 10%)<br><br>Canabinol (CBN) e Canabigerol (CBG): Não informado	CBD: 1,75% p/V<br>CBDA: 0,02% p/V<br>THC: 9,96% p/V<br>CBN: 0,12% p/V<br>CBG: 0,89% p/V<br>THCA: Não detectado	N.A.	HPLPC (POP.401)
Densidade[2]	Não informado	0,9613 g/mL	N.A.	Método Geral (POP.264)
Determinação do Teor de Peróxido por Titulação[2]	Não informado	0,98 mEq/Kg	N.A.	Titulação (POP.086)
Determinação de Solventes Residuais por GC-Headspace – Etanol[1]	Máx. 3000 ppm	2461,61 ppm	133,30 ppm	GC-Headspace (POP.326)

LQ – Limite de Quantificação / LD – Limite de Detecção / N.A. – Não Aplicável

1. Referência: Não Informada
2. Referência: Farm. Bras. VI

GUSTAVO BERTOL:04128423947 Assinado de forma digital por GUSTAVO BERTOL:04128423947 Dados: 2025.09.18 16:44:35 -03'00'

Gustavo Bertol

Certificado de Análise

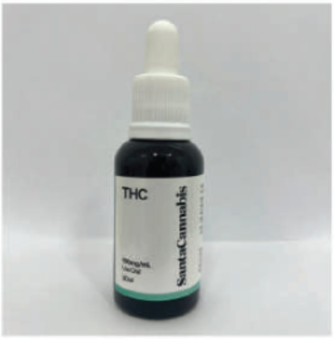

Extrato de Cannabis sativa padronizado em 100 mg/mL de Tetrahidrocanabinol - 30 mL

LABORATÓRIO SUBCONTRATADO: DALL PHYTOLAB
FABRICAÇÃO: 16/07/2025 - VALIDADE: 16/07/2026
LOTE: THC100001 - EMISSÃO DOS RESULTADOS: 04/09/2025

Ensaio	Método	Resultado
Canabinoides	HPLC	Aprovado
Aspecto	Método Geral	Aprovado
Oxidação	Titulação	Aprovado
Metais Pesados	AAS-GF e AAS-HG	Aprovado
Solvente Residual	GC-Headspace	Aprovado
Microbiológico	Farm. Bras. VI	Aprovado

ENSAIO*	ESPECIFICAÇÃO	RESULTADO	LQ**	MÉTODO	STATUS
**CANABINÓIDES**					
Determinação do Teor de Canabinoides por HPLC[1]	CBD+CBDA: Não Informado<br>THC+THCA: 9% a 11% p/V<br>(10% ± 10%)	CBD: 1,75%<br>CBDA: 0,02%<br>THC: 9,96%<br>THCA: Não detectado		HPLC	**APROVADO**

CLIENTE: Associação Brasileira de Cannabis Medinal

ORÇAMENTO/PROTOCOLO: 2426.25

ENDEREÇO: Rua General Liberato Bittencourt, 1885 – Sala 504

AMOSTRA: Solução oleosa de Cannabis

LOTE: CBD100001

DATA DE FABRICAÇÃO: 07/07/2025

DATA DE VALIDADE: 07/07/2026

AMOSTRAGEM: Realizada pelo cliente

DATA DE RECEBIMENTO: 04/08/2025

REALIZAÇÃO DOS ENSAIOS: 21/08/2025

EMISSÃO DOS RESULTADOS: 04/09/2025

RESULTADOS

Ensaio	Especificação do Cliente	Resultado	LQ	Método
Determinação do Teor de Canabinoides por HPLC[1]	Canabidiol (CBD + CBDA): 9 a 11% p/V (10,0 ± 10%)<br><br>Tetrahidrocanabidiol (THC + THCA): 0,45% a 0,55% p/V (0,1 ± 10%)<br><br>Canabidiol (CBN) e Canabigerol (CBG): Não informado	CBD: 9,35% p/V<br>CBDA: 0,19% p/V<br>THC: 0,41% p/V<br>CBN: Não detectado<br>CBG: 0,22% p/V<br>THCA: Não detectado	N.A.	HPLPC (POP.401)
Densidade[2]	Não informado	0,9547 g/mL	N.A.	Método Geral (POP.264)
Determinação do Teor de Peróxido por Titulação[2]	Não informado	2,40 mEq/Kg	N.A.	Método Geral (POP.086)
Determinação de Solventes Residuais por GC-Headspace – Etanol[1]	Máx. 13.000 ppm	1254,12 ppm	133,30 ppm	GC-Headspace (POP.326)

LQ – Limite de Quantificação / LD – Limite de Detecção / N.A. – Não Aplicável

1. Referência: Não Informada
2. Referência: Farm. Bras. VI

GUSTAVO BERTOL:04128423947

Assinado de forma digital por GUSTAVO BERTOL:04128423947
Dados: 2025.09.04 16:26:41 -03'00'

Gustavo Bertol

Certificado de Análise

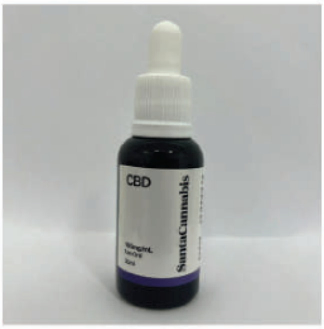

Extrato de Cannabis sativa padronizado em 100 mg/mL de Canabidiol - 30 mL

LABORATÓRIO SUBCONTRATADO: DALL PHYTOLAB
FABRICAÇÃO: 07/07/2025 - VALIDADE: 07/07/2026
LOTE: CBD100001 - EMISSÃO DOS RESULTADOS: 04/09/2025

Ensaio	Método	Resultado
Canabinoides	HPLC	Aprovado
Aspecto	Método Geral	Aprovado
Oxidação	Titulação	Aprovado
Metais Pesados	AAS-GF e AAS-HG	Aprovado
Solvente Residual	GC-Headspace	Aprovado
Microbiológico	Farm. Bras. VI	Aprovado

ENSAIO*	ESPECIFICAÇÃO	RESULTADO	LQ**	MÉTODO	STATUS
CANABINÓIDES					
Determinação do Teor de Canabinoides por HPLC[1]	CBD+CBDA: 9% a 11% p/V (10,0% ± 10%)<br>THC+THCA: 0,45% a 0,55% p/V (0,5% ± 10%)	CBD: 9,35%<br>CBDA: 0,19%<br>THC: 0,41%<br>THCA: Não detectado		HPLC	APROVADO
