# Supplementary material for: Case Report: Oral and topical chronic administration of THC-rich and CBD-rich cannabis oil as palliative care in a rescued horse with open wound, sarcoid and chronic pain
Source: Front Vet Sci. 2026 Jun 2;13:1794084. doi: 10.3389/fvets.2026.1794084 (PMC13269266; doi:10.3389/fvets.2026.1794084)
Supplement: SUPPLEMENTARY MATERIAL 2 — Radiographic (Supplementary Figure 1) and histopathologic diagnosis (Supplementary Figure 2); Longitudinal comparison of hemogram and biochemical parameters (Supplementary Figure 3). [file Data_Sheet_2.pdf]

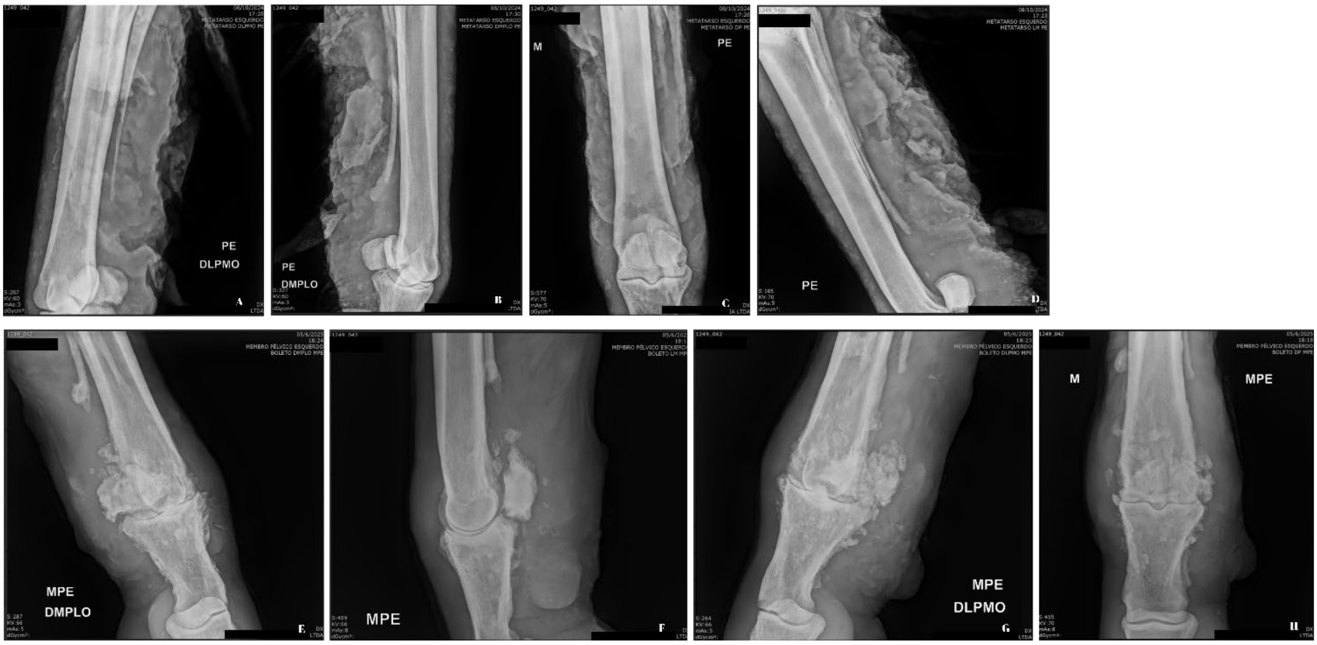

Figure 1 - Study of the left hind limb (metatarsus and fetlock) was performed in four standard views (lateral-medial, dorsoplantar, dorsomedial–plantarolateral oblique [DMPLO], and dorsolateral–plantaromedial oblique [DLPMO]). Radiographic evaluation of the distal pelvic limb during rescue and subsequent diagnosis. (A–D) Radiographs obtained at the time of rescue, performed to assess soft tissue involvement and joint integrity of the distal limb. Marked soft tissue alterations are evident. In the lateral-medial and DLPMO view there is mild cortical irregularity and subperiosteal reaction along the caudal cortex of the third metatarsal bone, which may indicate the early development of osteomyelitis or a reactive bone response due to chronic inflammation or local tissue invasion. (E–H) Follow-up radiographs obtained at the time of diagnosis, demonstrating osteomyelitis with primary involvement of the sesamoid bones, characterized by irregular bone margins, periosteal reaction, and adjacent soft tissue changes.

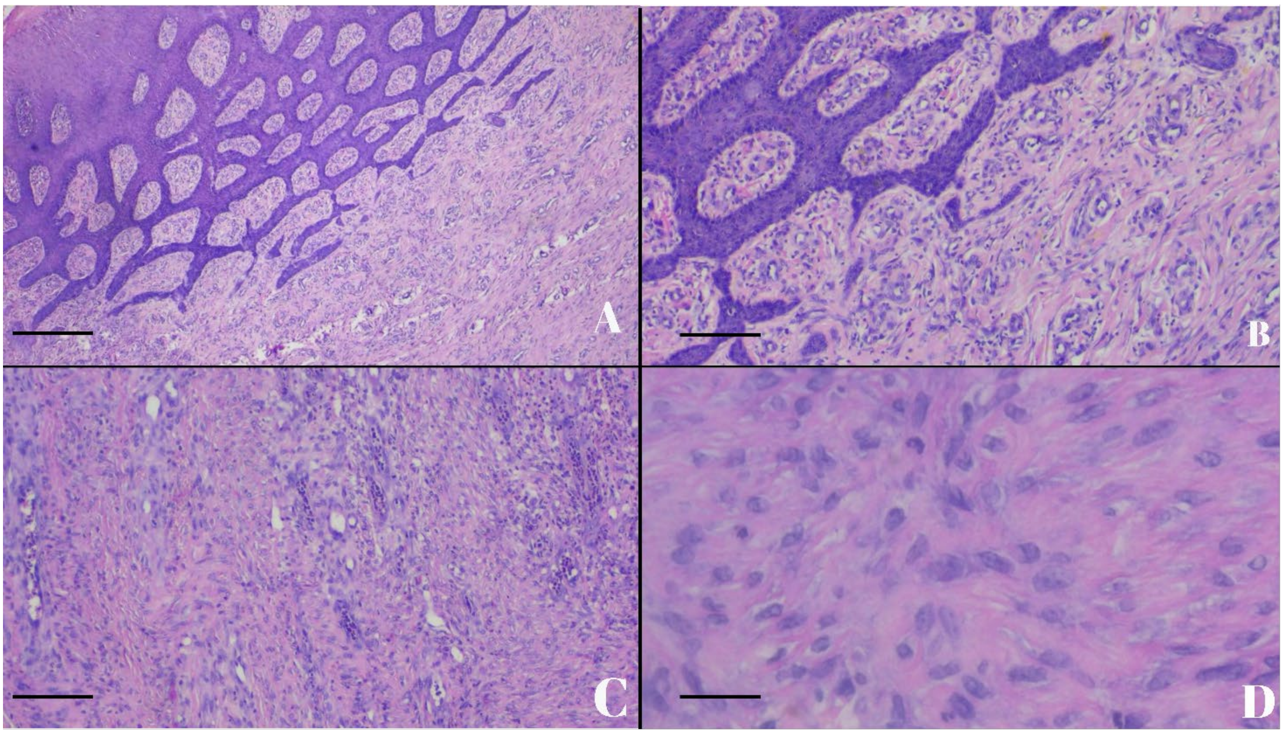

Figure 2 - Hematoxylin and eosin (H&E)–stained histological sections from different tissue fragments of an equine sarcoid. (A) Low-power view (10×) showing epidermal hyperplasia with characteristic *picket-fence* (palisading) arrangement of dermal fibroblasts beneath the epidermis. (B) High-power view (40×) highlighting pronounced epidermal–dermal interaction and well-defined fibroblastic proliferation consistent with sarcoid architecture. (C) intermediate magnification (20×) demonstrating densely packed, interwoven collagen undles and spindle-shaped fibroblasts arranged in a whorled pattern within the dermis. (D) High-power detail (100×) showing neoplastic spindle cells with elongated nuclei embedded within a collagen-rich stroma. Scale bar: 50 µm.

Parameter	Before (18/10/24)	During (31/05/25)	Reference Range
Erythrocytes (million/μL)	2.77	7.08	6.0 – 12.0
Hemoglobin (g/dL)	5.1	11.1	8.0 – 14.0
Hematocrit (%)	15	31	30 – 48
MCV (fL)	54	43.79	34 – 58
MCH (pg)	18	15.68	13 – 19
MCHC (%)	34	35.81	31 – 37
RDW (%)	18	18.7	18 – 22
Platelets (thousand/μL)	265	131	100 – 500
Leukocytes (thousand/μL)	9.0	9.3	6.0 – 12.0
Neutrophils (/μL)	7200	5022	2100 – 9000
Lymphocytes (/μL)	1530	3534	1500 – 6000
Monocytes (/μL)	270	651	100 – 1000
Eosinophils (/μL)	0	93	100 – 1000
Basophils (/μL)	0	0	0 – 250
Total Plasma Protein (g/dL)	7.3	8.0	6.0 – 8.5

Parameter	Before	During	Reference Range
ALT (U/L)	5	13	3 – 25
AST (U/L)	228	392	94 – 309
Alkaline Phosphatase (U/L)	210	181	143 – 395
Urea (mg/dL)	29	35	21 – 55
GGT (U/L)	—	34	12 – 45
Total Protein (g/dL)	—	7.6	5.2 – 7.9
Albumin (g/dL)	—	3.4	2.6 – 3.7
Globulins (g/dL)	—	4.2	2.6 – 4.0
Glucose (mg/dL)	—	108	75 – 115
Creatinine (mg/dL)	—	1.23	1.2 – 1.9
Triglycerides (mg/dL)	—	25	4 – 44
Total Cholesterol (mg/dL)	—	118	75 – 150

Figure 3 - Hematological and serum biochemical parameters obtained before clinical intervention (18 October 2024) and during follow-up (31 May 2025). The CBC revealed moderate non-regenerative anemia, characterized by low erythrocyte count, hemoglobin, and hematocrit. Despite this, RBC indices (MCV, MCH, MCHC) were within or near reference, suggesting normocytic, normochromic anemia. Neutrophilia and mild lymphopenia were consistent with stress leukogram or chronic inflammation. Platelet count and plasma protein were within normal limits. Liver enzymes (AST, ALT) and alkaline phosphatase were within normal limits. AST elevation may reflect muscle catabolism or tissue injury rather than hepatic dysfunction, which is consistent with the patient's severe muscle atrophy and chronic tissue damage. Values are presented alongside established equine reference ranges. The table summarizes changes in red and white blood cell indices, platelet count, and selected biochemical markers, allowing longitudinal assessment of hematologic recovery and systemic metabolic status throughout clinical management.
